# A Fetal ECG Monitoring System Based on the Android Smartphone

**DOI:** 10.3390/s19030446

**Published:** 2019-01-22

**Authors:** Li Yuan, Yanchao Yuan, Zhuhuang Zhou, Yanping Bai, Shuicai Wu

**Affiliations:** College of Life Science and Bioengineering, Beijing University of Technology, Beijing 100124, China; 18811712052@163.com (L.Y.); yuanyc798@163.com (Y.Y.); zhouzh@bjut.edu.cn (Z.Z.); baiyanping@bjut.edu.cn (Y.B.)

**Keywords:** fetal electrocardiogram (ECG), wearable ECG collector, non-invasive monitoring, Android smartphone, fast fixed-point algorithm for independent component analysis (FastICA)

## Abstract

In this paper, a fetal electrocardiogram (ECG) monitoring system based on the Android smartphone was proposed. We designed a portable low-power fetal ECG collector, which collected maternal abdominal ECG signals in real time. The ECG data were sent to a smartphone client via Bluetooth. Smartphone app software was developed based on the Android system. The app integrated the fast fixed-point algorithm for independent component analysis (FastICA) and the sample entropy algorithm, for the sake of real-time extraction of fetal ECG signals from the maternal abdominal ECG signals. The fetal heart rate was computed using the extracted fetal ECG signals. Experimental results showed that the FastICA algorithm can extract a clear fetal ECG, and the sample entropy can correctly determine the channel where the fetal ECG is located. The proposed fetal ECG monitoring system may be feasible for non-invasive, real-time monitoring of fetal ECGs.

## 1. Introduction

Cognitive and exercise impairment caused by fetal hypoxia during the perinatal period remains a serious health problem worldwide [1]. Physicians track fetal growth and development during the perinatal period by monitoring changes in of heart sounds, heart rate, and fetal electrocardiogram (ECG). The fetal ECG reflects the electrophysiological activity of the fetal heart. Using fetal ECG waveform analysis, fetal abnormalities during fetal development, such as fetal distress and intrauterine hypoxia, may be detected.

Currently, there are two ways to obtain a fetal ECG. One is the invasive scalp electrode method, which can directly measure the pure fetal ECG signal. However, it can only detect fetal ECG signal during birth, and because it is invasive it may cause harm to the mother and the fetus. Another method is the non-invasive abdominal electrode method. Signals from the abdominal body surface are collected by placing an electrode patch in the abdomen of the mother, which allows for long-term monitoring during pregnancy. However, the signals from the maternal abdominal surface are very complex, containing not only maternal ECG and weak fetal ECG signals, but also the mother’s respiratory noise, frequency interference, and other signals [2]. In particular, the magnitude of the maternal ECG detected in the abdomen is about 2–10 times that of the fetal ECG [3], which makes the extraction of the fetal ECG difficult. Therefore, it is necessary to develop a non-invasive method that can extract the fetal ECG effectively.

At present, fetal ECG extraction algorithms mainly include adaptive filtering [4,5], wavelet analysis [6], matched filtering [7], blind source separation (BSS) [8,9], independent component analysis (ICA) [10], neural network [11], and singular value decomposition (SVD) [12]. Among these methods, ICA can separate the source signals from the mixed signals under the assumption that the source signals are statistically independent of each other, without needing any information regarding the source signals or the mixed matrix. Therefore, ICA is considered a promising method for extracting fetal ECGs. Because of its fast convergence, the fast fixed-point algorithm for independent component analysis (FastICA) [13] has been widely used in the extraction of fetal ECGs. Therefore, this paper uses FastICA algorithm based on negative entropy maximization to extract fetal ECGs.

With the development of electronic and communication technology, smartphones have been popularized and are now in millions of households. To realize home-based fetal ECG monitoring, this paper proposes a fetal ECG monitoring system based on smartphone technology. Three-lead abdominal ECG signals are collected by a wearable fetal ECG collector, and then the collected abdominal signals are sent to a smartphone app via Bluetooth. The smartphone app pre-processes the three-channel abdominal ECG signal and uses the FastICA algorithm to separate the source components. Then, the smartphone app uses the sample entropy to detect the fetal ECG signal and calculates the fetal heart rate. Finally, the fetal ECG waveform and fetal heart rate are displayed in real time on the smartphone interface, and a warning is given when the heart rate is abnormal.

## 2. Materials and Methods

**Figure 1:**
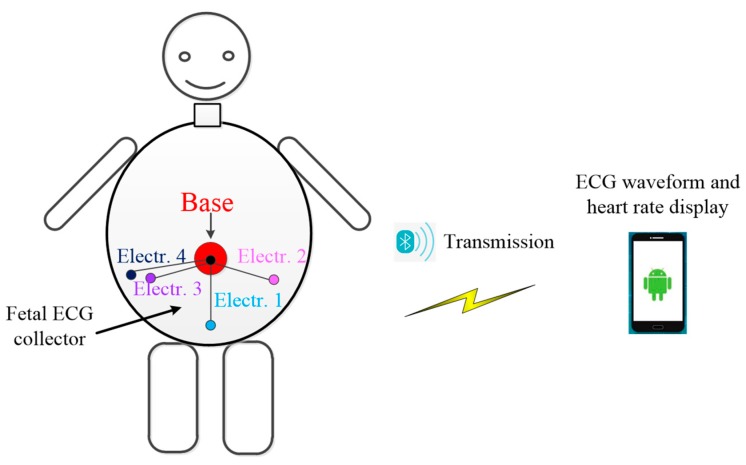
Illustration of the fetal ECG monitoring system.

The fetal ECG monitoring system is illustrated in Figure 1. We designed a three-lead fetal ECG collector. Five electrodes were used to collect three-channel abdominal ECG signals from pregnant women. The five electrodes were pasted to the abdomen of pregnant women according to [14]. The pasting method was simple and easy to learn. The collected three-lead abdominal ECG signals were transmitted to the smartphone through Bluetooth. App software was developed based on the smartphone in order to extract fetal ECG signals in real time. The extracted fetal ECG was displayed on the smartphone screen. A warning was given for abnormal fetal heart rate.

### 2.1. Hardware Design of Fetal ECG Collector

**Figure 2:**
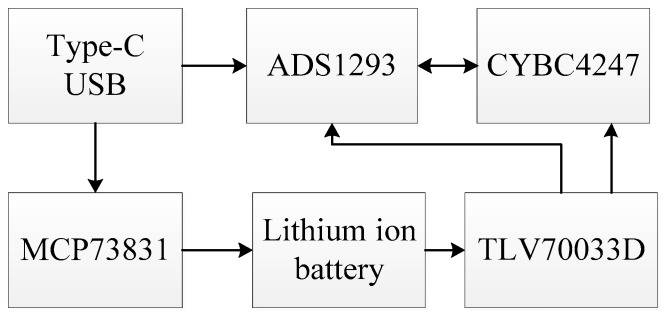
Block diagram of the fetal ECG collector circuit.

The structure of the fetal ECG collector is shown in Figure 2. It mainly consisted of three modules, a processor with Bluetooth 4.1, a signal acquisition front end, and a low power supply module. The processor, CY8C4247 (Cypress Semiconductor, San Jose, CA, USA), was an ARM-based Cortex-M0 core programmable embedded system controller (32 bit, 48 MHz). The Bluetooth module was integrated in the processor. The signal acquisition front end, ADS1293 (Texas Instruments, Dallas, TX, USA), was a three-channel, 24-bit integrated analog front end for biopotential measurement. Each channel could be set for a specific sampling rate and bandwidth, with AC/DC electrode dropout detection. The low power supply module consisted of a charging chip MCP73831 (Microchip Technology, Chandler, AZ, USA) and a 3.3 V regulator chip TLV70033D (Texas Instruments, Dallas, TX, USA). It was used to charge the lithium-ion battery and supply power to the fetal ECG monitoring system circuit after 3.3 V regulation. The system stopped working when charging. When the USB lead wire was separated from the collecting end, the ECG acquisition module worked with low power consumption and no longer sent data to the smartphone. When the USB lead wire was in contact with the acquisition end, the ECG acquisition module started to work. There were anti-aliasing filters in ADS1293. For example, the electromagnetic interference low-pass filtering circuit could filter out common-mode interference and high-frequency signal interference. After ECG signal amplification, a band-pass filter filtered out interference signals. There was also an integrated right leg drive circuit. The programmable cut-off frequency of the anti-aliasing filters in ADS1293 ranged from 5 to 1280 Hz. The frequency of the fetal ECG is generally 0.03–100 Hz. As the sampling frequency was 250 Hz, the cut-off frequency of the anti-aliasing filter was set to 110 Hz in this study, in accordance with the Nyquist sampling theorem. Hence, the bandwidth was 0–110 Hz.

**Figure 3:**
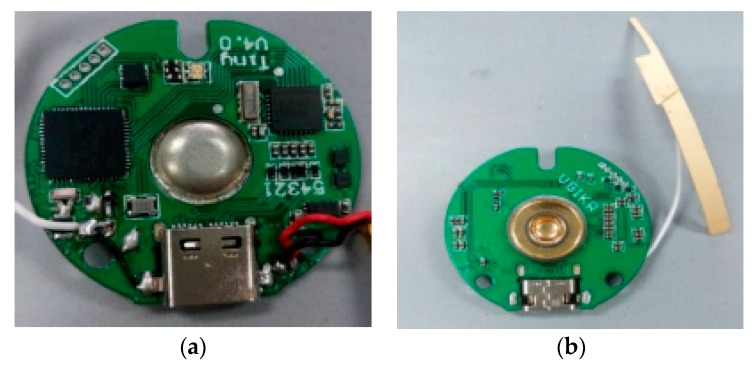
(**a**) Front and (**b**) back sides of the fetal ECG collector circuit board.

**Figure 4:**
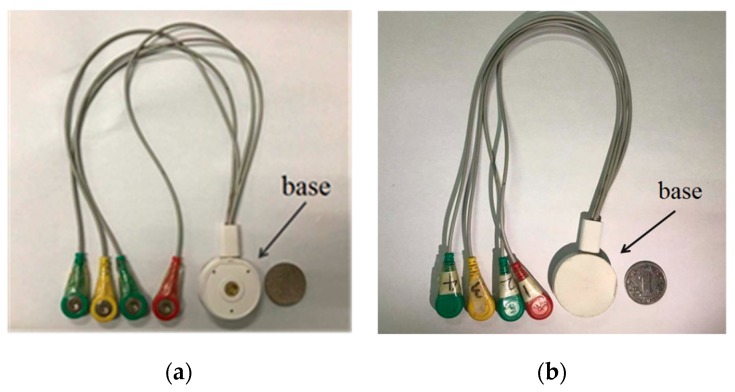
Fetal ECG collector: (**a**) Front view; (**b**) back view.

The fetal ECG collector circuit board is shown in Figure 3. Four electrodes were used for the fetal ECG collector, as shown in Figure 4.

### 2.2. Fetal ECG Extraction Algorithm

**Figure 5:**
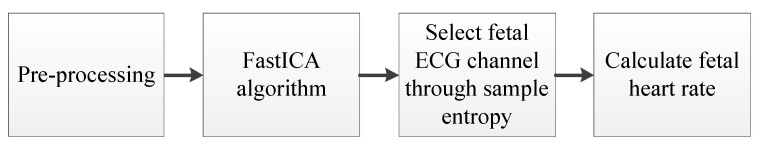
Flow chart of fetal ECG extraction.

The signal collected by the fetal ECG collector was a mixed ECG signal from the mother and the fetus. The maternal ECG amplitude is generally 2–10 times that of the fetus. Clifford et al. demonstrated that there are both temporal and frequency overlaps between some fetal QRS complexes and the maternal QRS [15]. The FastICA algorithm is a method for real-time fetal ECG extraction. FastICA is an improvement on ICA and is a fast optimization iterative algorithm for processing high-dimensional data. However, the channel position of each source component separated by FastICA needs to be determined, and it is necessary to visually observe the channel where the fetal ECG signal is located, in order to perform subsequent fetal heart rate calculation. To address this issue, we employed sample entropy to process the signal after FastICA separation, automatically selecting the channel where the fetal ECG signal is located. For a time series signal, sample entropy identifies similar epochs and computes a non-negative value for the signal; a larger value of sample entropy corresponds to more irregularity in the signal [16]. Finally, the fetal heart rate was computed. The flow chart of the fetal ECG extraction algorithm is shown in Figure 5.

#### 2.2.1. Abdominal Signal Pre-Processing

The pre-processing included baseline drift cancellation, mean value cancellation, and whitening. The baseline signal of each channel was estimated by a third-order low-pass Butterworth filter with a cut-off frequency of 5 Hz [2]. The baseline drift cancelled signal was obtained by subtracting the estimated baseline signal from the observed signal. The mean value cancellation [Equation (1)] was to reduce the computational complexity of the FastICA algorithm.

(1) $$X_{2}=X_{1}-E\{X_{1}\},$$

where $X_{2}$ was the mean value cancelled signal, *X*_1_ was the baseline drift cancelled signal, and *E*{.} was the expectation operator. The whitening [Equation (2)] is to remove the correlation between signals and make the signal second-order independent.

(2) X=ED−12E,

where *X* was the whitened signal, and *E* and *D* were the eigenvalues and eigenvectors of the covariance matrix of *X*_2_, respectively.

#### 2.2.2. FastICA Algorithm

The FastICA algorithm is an algorithm based on fixed-point iteration [17], which aims to make *y* = *w^T^X* have the largest non-Gaussianity, where *w* is a row of the separation matrix *W*. Non-Gaussianity serves as a proxy for statistical independence. Equation (3) was used as the objective function:

(3) $$J(y)\approx {\left\{E[G(y)]-E[G(v)]\right\}}^{2},$$

where *v* was a Gaussian random variable with zero mean and unit variance. It was assumed that *y* also had zero mean and unit variance. *G*(.) was a nonquadratic function. Since the maternal abdominal mixed signal is a sub-Gaussian signal, we selected *G*(.) = *y*^4^/4. Sub-Gaussian signals have a fourth-order cumulant of kurtosis that is smaller than zero [18].

According to the Kuhn–Tucker condition, Equation (4) could be obtained:

(4) $$E\{Xg(w^{T}X)\}-\beta w=0,$$

where *β* was a constant defined by $\beta =E\{w_{0}{}^{T}Xg(w_{0}{}^{T}X)\}$; $w_{0}$ was the initial value of *w*; and *g*(.) was a nonlinear function and was the derivative of *G*(.). The Newton iterative method was employed to solve Equation (4). The left part of Equation (4) was denoted as *F*(*w*), and the Jacobian matrix *JF*(*w*) was

(5) $$JF(w)=E\{XX^{T}g^{\prime }(w^{T}X)\}-\beta I.$$

Because the data were whitened, Equation (5) could be simplified as

(6) $$E\{XX^{T}g^{\prime }(w^{T}X)\}\approx E\{g'(w^{T}X)\}I.$$

The Jacobin matrix was a diagonal matrix, and its inverse matrix could be simply calculated. Similarly, the value of $w_{0}$ was replaced with the current value of *w* for the constant *β*. Therefore, we could obtain the approximated Newton iterative equation:

(7) $$w_{k+1}=w_{k}-\frac{\left[E\{Xg(w_{k}^{T}X)\}-\beta w_{k}\right]}{\left[E\{g^{\prime }(w_{k}^{T}X)\}-\beta \right]},$$

where $\beta =E\{w^{T}Xg(w^{T}X)\}$, and $w_{k+1}$ represented the updated value of $w_{k}$. In order to improve the stability of the algorithm, *w* was normalized by $w_{k+1}=w_{k+1}/\left\|w_{k+1}\right\|$ after iteration. To simplify Equation (7), we obtained an iterative equation for the simplified FastICA algorithm:

(8) $$w_{k+1}=E\{Xg(w_{k}^{T}X)\}-E\{g^{\prime }(w_{k}^{T}X)\}w_{k}.$$

#### 2.2.3. Sample Entropy

Sample entropy is a measure of the irregularity of a time series signal [16]. A larger value of sample entropy indicates a higher irregularity of the signal [16]. From our experiments, it was found that, after the separation of the three source components by FastICA, the source component of the fetal ECG had the largest value of sample entropy. Thus, we speculated that the source component of the fetal ECG had the largest irregularity. On the basis of this finding, the sample entropy was used to determine the channel where the fetal ECG signal was located, and then the fetal heart rate was calculated.

For the ECG signal sequence *u*(*k*) (1 ≤ *k* ≤ *N*), *N*—*m* vectors, $X_{i}^{m}$ = {*u*(*i*), *u*(*I* + 1), ..., *u*(*i* + *m* − 1)}, were formed, where 1 ≤ *i* ≤ *N* − *m*, and $X_{i}^{m}$ were sequences of *m* time points from data points *u*(*i*) to *u*(*i* + *m* − 1). The distance between two vectors, $X_{i}^{m}$ and $X_{j}^{m}$, was defined as

(9) $$d_{i,j}^{m}=d[X_{i}^{m},X_{j}^{m}]=\max_{k=0}^{m-1}|u(i+k)-u(j+k).$$

Given a threshold *r*, for each *i* ≤ *N* − *m*, $B_{i}^{m}(r)$ was defined as

(10) $$B_{i}^{m}(r)=N^{m}(i)/(N-m-1),$$

where $N^{m}(i)$ denoted the number of $d_{i,j}^{m}$ that was smaller than threshold *r*.

Similarly, $A_{i}^{m+1}(r)$ was defined as

(11) $$A_{i}^{m+1}(r)=N^{m+1}(i)/(N-m-1),$$

where $N^{m+1}(i)$ denoted the number of $d_{i,j}^{m}$ that was smaller than threshold *r*, when the dimension was increased to *m* + 1 data points.

Then, the sample entropy was calculated by

(12) $$SampEn(m,r,N)=-\ln(\sum_{i-1}^{N-m}A_{i}^{m+1}(r)/\sum_{i-1}^{N-m}B_{i}^{m}(r)).$$

### 2.3. Android Smartphone App Software Design

We designed the fetal ECG monitoring system based on the Android smartphone, using low-power Bluetooth to communicate with the fetal ECG collector. We designed a data package and a data transfer protocol for transmission. Each package contained 146 bytes. The smartphone parsed the received data using the data transfer protocol. From a data package, 45 data points were parsed, corresponding to 15 data points per channel. The data transmitted from the collector were received and processed, and fetal ECG signals were separated and extracted. 

**Figure 6:**
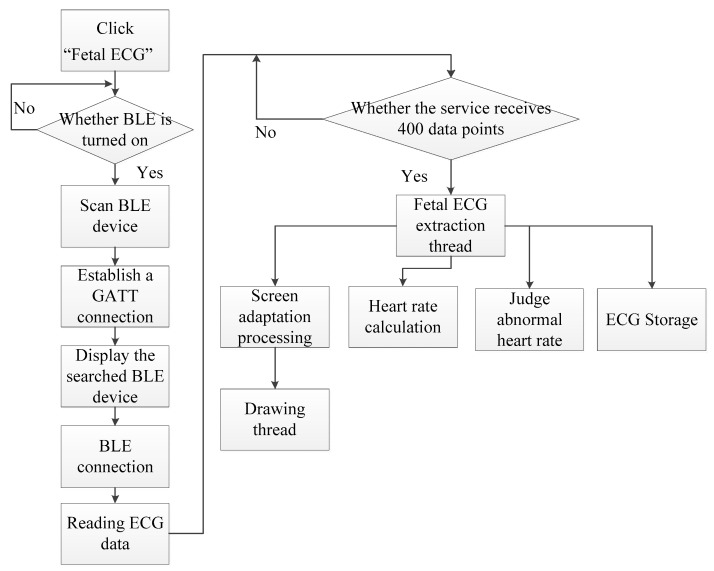
Flow chart of the Android app software.

The flow chart of the Android app software is shown in Figure 6. By setting up the service, the app started preparing (turning on Bluetooth) to scan the Bluetooth Low Energy (BLE) device and established a GATT (Generic Attribute Profile) connection via the address. After the connection was accomplished, the smartphone sent a start command (0X01, 0X00) to the processor, CY8C4247. After receiving the command, the processor sent 15 maternal abdominal ECG data points (for each of the three channels) to the smartphone. Then, the app performed real-time filtering (removing baseline drift and high frequency interference). When the service received 400 data points, these data were transferred to the fetal ECG extraction thread. Then, FastICA was performed for fetal ECG extraction, and the sample entropy was conducted to identify the channel where the fetal ECG signal was located. The fetal heart rate was calculated and displayed. Besides local storage, the fetal ECG data could be uploaded to the cloud.

Table 1 shows the app software modules, including a registration/login module, a communication module, a display module, a data processing module, a storage module, and a cloud interaction module.

**Figure 7:**
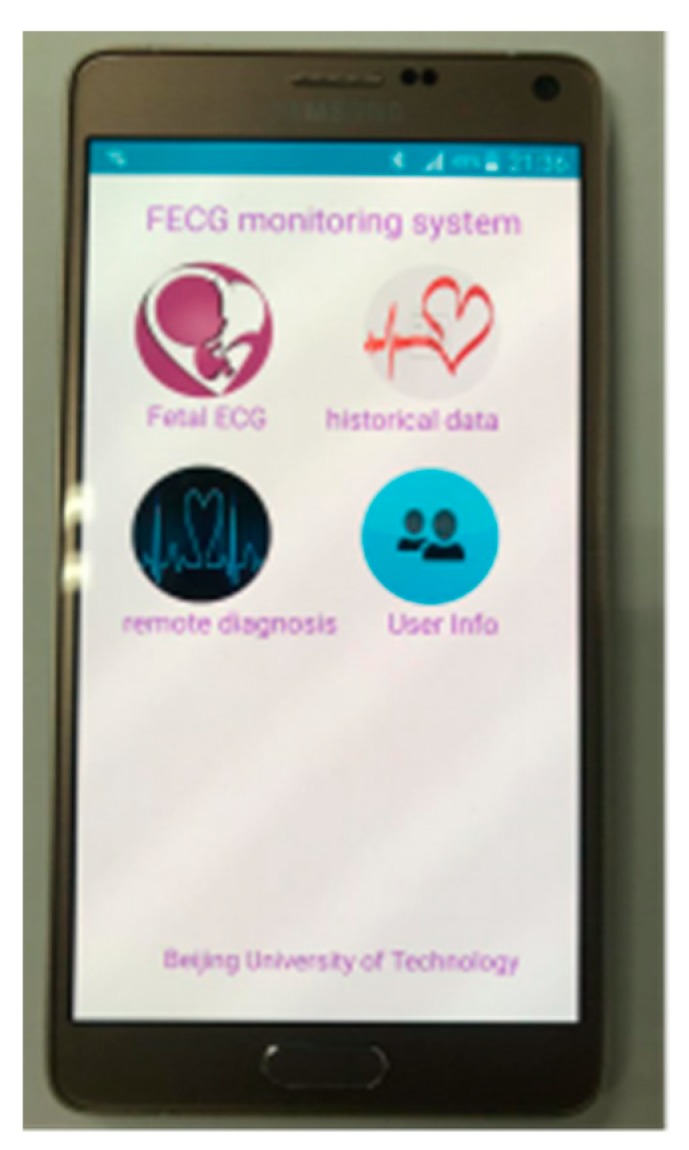
Android phone client main interface.

The main interface of the app is shown in Figure 7. The “fetal ECG” was used for displaying, in real time, fetal ECG waveforms and heart rates. The “historical data” was used for viewing the saved fetal ECG data. The “remote diagnosis” was used for uploading fetal ECG data to the cloud, and also for receiving guidance and advice from clinicians. Through the “user info”, the age, gestational age, physical condition, and other information about the pregnant woman could be viewed.

## 3. Results

### 3.1. Algorithm Verification

**Figure 8:**
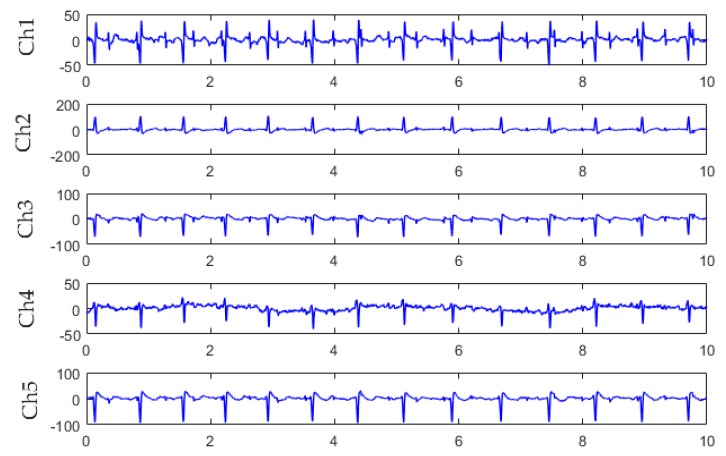
Five-channel abdominal signals from the DaIsy database.

We used the DaIsy (Database for the Identification of Systems) database [19] for algorithm verification. The DaIsy database contained ECG data collected from the abdomen of pregnant women through electrodes. The sampling frequency was 250 Hz, and the acquisition time was 10 s. Figure 8 shows the original five-channel abdominal signals in the DaIsy database.

**Figure 9:**
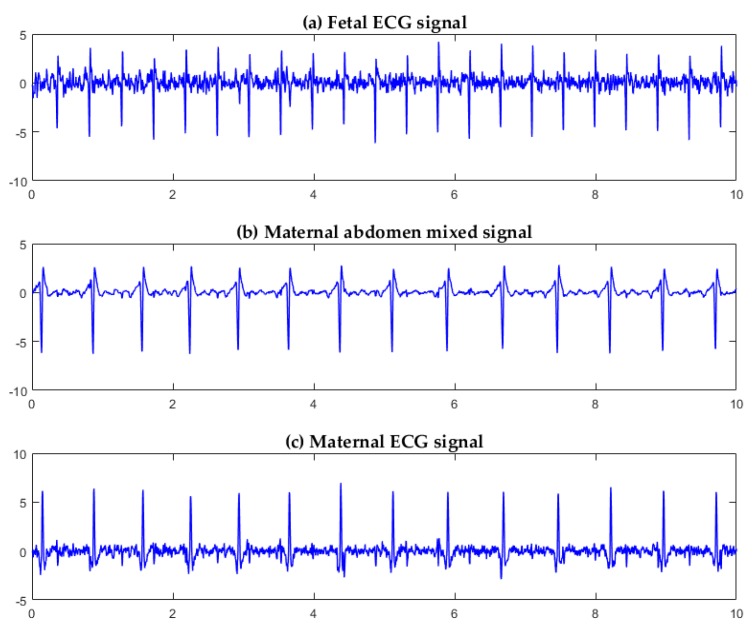
Three source component signals separated by the FastICA algorithm for Ch1, Ch3, and Ch4 signals from the pregnant woman (Figure 8): (**a**) fetal ECG signal; (**b**) maternal abdominal mixed signal; (**c**) maternal ECG signal.

Three channels of signals were selected from the five-channel DaIsy database for FastICA separation. Each channel corresponded to a different position on the maternal abdomen for signal collection. For Ch1, Ch3, and Ch4 signals from the pregnant woman (Figure 8), the three source component signals, separated by the FastICA algorithm, are shown in Figure 9. Figure 9a,b,c show the fetal ECG signal, the maternal abdomen mixed signal, and the maternal ECG signal, respectively. The fetal and maternal ECG signals had similar morphology, but the fetal heart beat frequency was higher than the maternal heart beat frequency. The sample entropy values of Figure 9a,b,c were 1.02, 0.35, and 0.61, respectively. The fetal ECG had the largest entropy value. 

**Figure 10:**
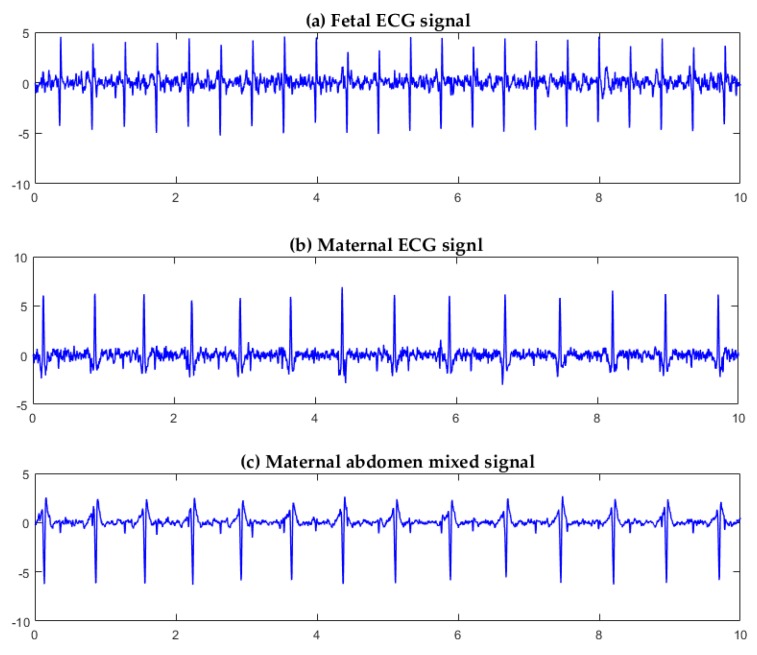
Three source component signals separated by the FastICA algorithm for Ch2, Ch3, and Ch5 signals from the pregnant woman (Figure 8): (**a**) fetal ECG signal; (**b**) maternal ECG signal; (**c**) maternal abdominal mixed signal.

For Ch2, Ch3, and Ch5 signals from the pregnant woman (Figure 8), the three source component signals, separated by the FastICA algorithm, are shown in Figure 10. Figure 10a,b,c show the fetal ECG signal, the maternal ECG signal, and the maternal abdomen mixed signal, respectively. The sample entropy values of Figure 10a,b,c were 1.12, 0.96, and 0.43, respectively. The fetal ECG had the largest entropy value.

It can be seen from Figure 9 and Figure 10 that FastICA can successfully separate the three source component signals: fetal ECG, maternal ECG, and maternal abdomen mixed ECG, but the channels in which they are located are random. 

Table 2 shows the sample entropy values of 10 groups of three-channel ECG data. Each group was sorted by channel number. The mean entropy values of fetal ECG, mixed ECG, and maternal ECG were 1.22, 0.29, and 0.73, respectively. The difference in entropy values is obvious, and the number of channels in which the fetal ECG signal is located can be automatically identified according to this feature, which is especially important in the real-time extraction and analysis of the fetal ECG.

### 3.2. Fetal ECG Collector Performance Testing

The fetal ECG collector designed in this work was tested for performance. The testing results are shown in Table 3. All the testing results met the requirements of the China National Standard. In particular, the minimum detectable signal of the collector was 2 μV, while the amplitude of fetal ECG signals is generally 10–50 μV. Moreover, the collector had a large input impedance and common-mode rejection ratio. The testing results indicated that the collector may collect ECG signals of desired quality and signal-to-noise ratio required by FastICA decomposition.

### 3.3. System Verification

**Figure 11:**
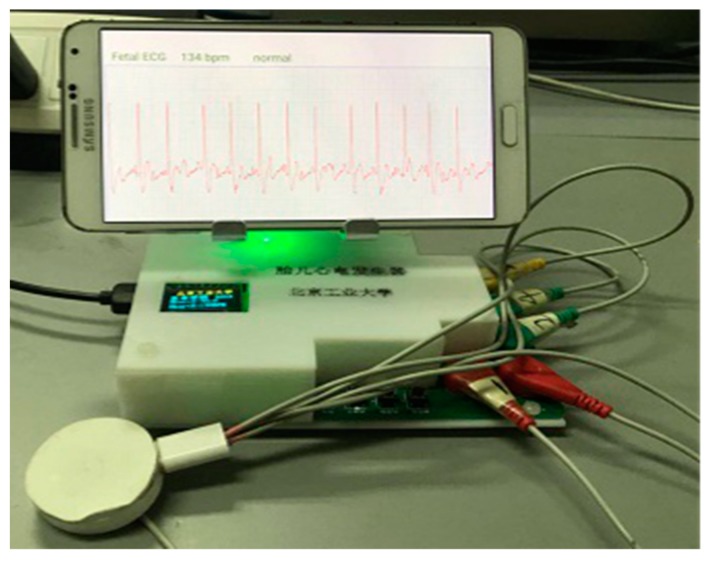
The fetal ECG monitoring system in operation.

In order to verify whether the system we developed can work normally, we used a maternal abdominal signal generator developed by our laboratory to simulate the abdominal surface signal of a pregnant woman, and used the fetal ECG collector to collect the three-channel abdominal surface signal. The maternal abdominal signal generator used MSP430F149 (Texas Instruments, Dallas, TX, USA) as the processor; the processor read the maternal abdominal ECG data (Ch1, Ch2, Ch3) and output three-channel ECG signals through digital-to-analog conversion. The three-lead abdominal signals were sent to the data receiving end of the smartphone app software, and the data processing module processed the received data. The processed fetal ECG and heart rate were displayed on the smartphone screen in real time. The time complexity of the proposed algorithm was *O*(*n*^2^). The smartphone used for testing was a Samsung Galaxy Note3 (Android OS 5.0, 2.3 GHz Qualcomm Snapdragon 800 quad-core processor, 3 GB memory). On receiving every 400 ECG data points (i.e., every 1.6 s, considering a sampling frequency of 250 Hz), the FastICA and sample entropy algorithms were called to extract fetal ECG signals. On the smartphone for testing, the fetal ECG extraction algorithm took approximately 1.5 s to process the 400 data points. Thus, there was a lag of ~1.5 s for the display of the fetal ECG. Using the maternal abdominal ECG signals simulated by the maternal abdominal signal generator, the system and algorithm proposed in this work can extract fetal ECGs for real-time monitoring (Figure 11). 

## 4. Discussion

In this paper, we designed a fetal ECG monitoring system based on the Android smartphone. The fetal ECG extraction algorithm was implemented on the smartphone app software to realize real-time display of fetal ECG waveform and fetal heart rate. Experimental results showed that the system may be feasible for real-time fetal ECG monitoring.

At present, clinicians often use ultrasound Doppler to detect fetal heart rate. Ultrasound diagnosis may stimulate fetal body movement and may cause harm to the fetus [20]. From the abdominal mixed ECG signal collected through the abdomen, the fetal ECG can be effectively characterized. This method is not only safe, but also more effective than ultrasound Doppler [21,22]. The wearable ECG collector we designed can provide long-term monitoring for pregnant women during the perinatal period, without causing harm to pregnant women or fetuses.

The three-lead ECG signal collector designed in this work can collect the maternal abdominal mixed signal, as shown in Figure 4. The collector was powered by a low-power lithium-ion battery that provided 24 h monitoring of the fetal ECG. At the same time, the fetal ECG monitoring system had a small number of electrodes, and the electrode sticking method was relatively simple, which is convenient for daily use by pregnant women. The signal received by the collector can be transmitted to the smartphone by Bluetooth. The smartphone app software can process the ECG data in real time, display the fetal ECG waveform and the fetal heart rate in real time on the screen of the smartphone, and automatically upload to the server when the fetal heart rate is abnormal. This may assist clinicians in taking appropriate measures to handle the abnormality.

Adaptive filtering [4,5] is a simple and fast method for fetal ECG extraction. However, the fetal ECG extracted using adaptive filtering is still affected by maternal ECG and other disturbances [23]. Wavelet-based methods [6] have shown promising performance for extracting the fetal ECG. However, appropriate selection of the mother wavelet is critical for the fetal and maternal ECG delineation. Neural network-based methods are limited by generalization, structure design, and local extremum [24]. Traditional BSS methods only deal with steady non-Gaussian signals, and their robustness is limited when noise exists [25]. ICA is a new BSS method with higher convergence and robustness, and has been widely used in biomedical signal processing. The FastICA algorithm is the most frequently used linear ICA method. FastICA is based on approximate negentropy and Newton iteration that reduces the computation. FastICA has shown the promising properties of easy implementation and fast convergence. However, the channels, in which the source components separated by FastICA are located, are not fixed (Figure 9 and Figure 10). To this end, we incorporated sample entropy with FastICA in this work. The experimental results showed that the source component of the fetal ECG had the largest sample entropy, corresponding to the largest irregularity [16], as can be observed in Figure 9 and Figure 10.

The Monica AN24 Wireless Fetal Monitoring System [26] monitors the fetal heart rate in real time and sends the fetal heart rate to the display device via Bluetooth. The disadvantage is that it can only display the fetal heart rate, but cannot display the ECG waveform, so much important fetal ECG information cannot be effectively displayed. Ali [27] proposed using an ECG collector to obtain the maternal abdominal signals, and then transmitting the signals to the smartphone through Bluetooth. The smartphone would then upload the signals to the base station by using General Packet Radio Service (GPRS). However, Ali [27] only built the framework of the remote monitoring system, and did not realize real-time monitoring of fetal ECGs. 

Another contribution of this work is the development of real-time monitoring of fetal ECGs based on a smartphone. Pregnant women can use the portable collector to collect abdominal mixed signals anytime and anywhere. The smartphone can display the fetal ECG waveform and fetal heart rate in real time. When the fetal ECG is abnormal, the abnormality is automatically uploaded to the cloud platform for further diagnosis by the clinician. The ST segment ECG of the fetus represents the energy-consuming process of ventricular repolarization and contains information about fetal hypoxia and distress [28]. However, the ST segment of the fetal ECG signal extracted by the algorithm was seriously polluted by noise. This limitation will be overcome in future work. For classification of fetal ECG abnormalities, advanced signal processing techniques may be considered [29]. Another limitation of this work was that the proposed system was only tested using maternal abdominal ECG signals simulated by the maternal abdominal signal generator, as shown in Figure 11. Clinical studies may be conducted in future work.

## 5. Conclusions

This paper presented a fetal ECG monitoring system based on the Android smartphone. The FastICA algorithm was used to separate the signal of each source component, and then the sample entropy was used to identify the channel where the fetal ECG signal was located. The fetal heart rate was calculated, and the fetal ECG and fetal heart rate were displayed in real time. Experimental results showed that the FastICA algorithm can extract clear a fetal ECG, and the sample entropy can correctly identify the channel where the fetal ECG signal is located. The proposed fetal ECG monitoring system may be feasible for real-time fetal ECG monitoring.

## Figures and Tables

**Table 1:** App software modules.

Module	Functional Description
Registration/login module	User registration and login
Communication module	Communicate with the fetal ECG collector via Bluetooth to receive data in real time
Display module	Display real-time ECG waveform, heart rate, and analysis results on the smartphone
Data processing module	Using FastICA to separate source components, sample entropy to identify fetal ECG signals, and thresholding to calculate fetal heart rate
Storage module	Save fetal ECG data to “.txt” format locally
Cloud interaction	Upload fetal ECG data to the cloud

**Table 2:** Sample entropy value.

Abdomen Three-lead Signal	Entropy of Fetal ECG	Entropy of Mixed ECG	Entropy of Maternal ECG
Ch1, Ch2, Ch3	1.32	0.3	0.92
Ch1, Ch2, Ch4	1.10	0.29	0.47
Ch1, Ch2, Ch5	1.56	0.22	0.61
Ch1, Ch3, Ch4	1.02	0.35	0.61
Ch1, Ch3, Ch5	1.03	0.26	0.90
Ch1, Ch4, Ch5	1.14	0.26	0.61
Ch2, Ch3, Ch4	1.54	0.33	0.88
Ch2, Ch3, Ch5	1.12	0.43	0.96
Ch2, Ch4, Ch5	1.14	0.25	0.61
Ch3, Ch4, Ch5	1.27	0.25	0.75
Mean	1.22	0.29	0.73

**Table 3:** Fetal ECG collector performance testing results.

Index	Testing Result	Requirement by the China National Standard
Dynamic input range	403 mV	300 mV
Input resistance	500 MΩ	>10 MΩ
Common-mode rejection ratio	95 dB	>45 dB
Gain accuracy	0.0%	10%
System noise	7 μV	50 μV
Frequency response	−0.2 to 0.9 dB	−3 to 3 dB
Minimum detectable signal	2 μV	50 μV
Timing accuracy	1 s	≤30 s
Duration of monitoring	34 h	>24 h
